# Influence of anisotropic pressure on viscosity and electrorheology of diethylene glycol-based MgAl_2_O_4_ nanofluids

**DOI:** 10.1186/1556-276X-9-170

**Published:** 2014-04-08

**Authors:** Gaweł żyła, Joanna Grzywa, Adam Witek, Marian Cholewa

**Affiliations:** 1 Department of Physics, Rzeszów 35-905, Poland; 2 Institute of Ceramics and Building Materials, Warsaw 02-676, Poland

**Keywords:** Nanofluid, Nanosuspension, Rheology, Rheometry, Viscosity, Pressure, Electrorheology

## Abstract

The paper presents results of rheological experiments on viscosity under anisotropic pressure and in electric field of diethylene glycol-based MgAl_2_O_4_ nanofluids. Nanofluids have been prepared in a two-step method. The dynamic viscosity of nanofluids with various mass concentrations of nanoparticles was measured in the range of shear rates from 10 s ^−1^ to 1,000 s ^−1^ in constant temperature under the pressure of 7.5 MPa. In the second type of experiments, different values of the electric field up to 2,000 V/mm was used. Thixotropy structure of MgAl_2_O_4_-DG nanofluids has been studied in electrical field.

## Background

Nanofluids, suspensions of nanoparticles, are increasingly being used in various industrial [1,2] and medical applications [3].

Most of the industrial applications result from increased thermal conductivity, which was reported for the first time in the second half of the 90th twentieth century. Since then the announcement of the initial results of the measurement of thermal conductivity of these materials, researchers had been studying them very intensively [4-9]. A large number of papers on thermal conductivity of these materials have resulted in the formation of theoretical models of this issue [10-12].

Medical applications are possible thanks to the antibacterial behavior of certain types of nanoparticles [13,14]. The issue of using nanofluids was then reduced to produce and use as a drug nanosuspension. In case of this type of application of nanofluids, not the thermal conductivity but the rheological properties of suspension are the most important factors.

Thermal conductivity of nanofluids depends on nanoparticle properties including material type, shape [15], size [16], aggregation [17], concentration, and type of base fluid. This parameters have also an influence on rheological behavior of nanofluids [18,19].

Unfortunately, at the moment, there does not exist a coherent theoretical model of the rheological properties of nanofluids. There are works of Einstein [20] and many other scientists who have theoretically studied the viscosity of the suspension [21,22]; but because of the unique properties of nanoparticles, these models cannot always be used to describe the nanofluids. Mackay et al. [23] presented non-Einstein-like decrease in viscosity of nanofluids caused by nanoscale effects.

There are a variety of methods of preparation of dry nanoparticles [24-26] since there is easy access to these materials and ability to use them in the production of nanofluids which will result in the further dynamic development of this field. As the base liquid, water [18,27,28], ethylene glycol [7,29], diethylene glycol [30,31], and ethyl alcohol [32,33] are used.

Viscosity of liquid depends not only on the temperature and shear rate, but also on the pressure. Though the viscosity of the fluid decreases with increasing temperature, it generally increases with increasing pressure. The pressure exerted on the fluid causes the approach of the particles towards each other and the increase of the intermolecular interactions; therefore, the viscosity of the fluid rises. An increase of the viscosity is higher for the fluids with a more composite structure because it impedes the movement of the particles under pressure. Thus, the scale of the viscosity increase of the liquid with the pressure depends on the type of fluid. The use of low pressure causes a slight increase in the viscosity. Whereas this increment is significant at higher pressure, influence of the pressure on viscosity is almost directly proportional to the pressure from the atmospheric pressure up to 100 MPa. The enhancement of the pressure to about 100 MPa doubles the value of the viscosity of most of the organic liquids [34]. However, in the area of high pressure, the dependence of the viscosity on the pressure is not directly proportional.

In the case of lubricating oils, the viscosity under the pressure of 1,000 MPa may increase even up to 10^7^ times, in comparison to the viscosity under atmospheric pressure [35]. At pressure ranks of several thousands of MPa, the impact of the intermolecular repulsion is visible, and thus, a curve of increment of viscosity with increasing pressure asymptotically approaches to a constant value [34].

The exception is the impact of the pressure on the viscosity of water and aqueous solutions. With the increase of the pressure to about 100 MPa and over a temperature to about 30°C, the viscosity of water decreases. The viscosity of water increases until from the pressures reaching a value of above 100 MPa and 30°C. Schmelzer et al. [36] measured the viscosity of water in the pressure range of 0 to 100 MPa and at the temperature range of 0°C to 25°C. This experiment confirmed the unique properties of water viscosity.

Consideration of the viscosity of various types of liquids depending on the pressure is not only a theoretical issue, but has a large practical importance. Exact knowledge of the viscosity of water at various pressures is important in the interpretation of the impact of pressure on the heat transfer in the aqueous solutions, flow problems, and also on the electrical conductance of aqueous electrolytes [37,38].

Horne and Johnson [39] measured the effect of hydrostatic pressure on the viscosity of pure water in the pressure and temperature ranges of 1 to 2,000 kg/cm^3^ and 2°C to 20°C, respectively, with a rolling ball type of viscometer. Using the same kind of viscometer, Stanley and Baten [40] measured the viscosity of water at pressures of 0 to 1,406 kg/cm^3^ and over a temperature range of 2°C to 30°C. In turn, Först et al. [41] presented experimental data for the viscosity of water at high pressures of up to 700 MPa in the temperature range of −13°C to 20°C with two different types of viscometers.

Whereas, Grimes et al. [42] showed experimental data on the viscosity of aqueous KCl solutions over the pressure range of 0 to 30 MPa and the temperature range of 25°C to 150°C using the oscillating-disk viscometer. The change of viscosity with pressure is of particular relevance in the field of lubrication.

On the other hand, the knowledge on viscosity of hydrocarbon mixtures under high pressure is also significant in the petrochemical industry. Oliveira and Wakeham [43] measured the viscosity of five different liquid hydrocarbons at pressures of up to 250 MPa in the temperature range of 303 to 384 K with a vibrating-wire viscometer.

Further, in the study of dynamic properties of ions or solvent particles at high pressures, the viscosity measurements of electrolyte solutions are important. The high-pressure viscosity is also relevant for many processes involving polymer solutions. From the other side, viscosity measurements under high pressures are also needed to estimate the diffusion rate of the particles in a fluid.

Thermophysical properties of nanofluids are also studied by others researchers. Pastoriza-Gallego et al. [18,44] examined the volumetric behaviour and the viscosity of CuO and Al_2_O_3_ in water nanofluids. Experimental density measurements of CuO-water nanofluids were performed at the pressure range from atmospheric pressure to 45 MPa, and the temperature range of 283.15 to 323.15 K, with a 10-K step. In turn, density measurements of Al_2_O_3_-water nanofluids were executed at an atmospheric pressure of 25 MPa, and the temperatures of 283.15, 298.15, and 313.15 K. Additionally, the viscosity measurements at atmospheric pressure were carried out.

Cabaleiro et al. [45] also experimentally determined the influence of pressure on the density of TiO_2_-ethylene glycol nanofluids. It was found that the impact of particle size on density is slight, but it may not be ignored. On the other side, the variations in viscosity are significant thus must be taken into consideration for any practical application. For this reason, examination on the influence of pressure on viscosity of nanofluids may have great practical importance.

Electrorheology is a field of science which studies liquids, whose viscosity changes reversibly and continuously under the influence of an electric field. Therefore, the viscosity of electrorheological fluids changes under the impact of an applied voltage. The electrorheological fluid is a suspension of particles in a base fluid, and for this reason, the simplest explanation for the viscosity increase is to assume that under the influence of an electric field, the particles connect to each other to form an ordered chain, whose direction is consistent with the direction of the force field. It increases the flow resistance of the liquid phase.

Effect of increased viscosity is proportional to the electric field intensity. That phenomenon is reversible - after the resolution of the electric field, the liquid returns to its initial properties. The effect of ‘curing liquid’ under the influence of an electric field is also called the Winslow effect, after the name of the American inventor Willis Winslow who was the first researcher of this phenomenon, and published an article about it in 1949 [46]. ‘Winslow liquids’ were based on oil, which contained a suspension of starch, lime, gypsum, silicon dioxide, or carbon.

The current understanding of the microscopic phenomena is that it is believed to control the electrorheological effects, and the models used to describe macroscopic behavior is presented in the review of Parthasarathy and Klingenberg [47]. Additionally, Hao [48] described the physical backgrounds behind phenomenon of electrorheological fluids.

Due to their unique properties, electrorheological liquids are used as working fluids in various types of machinery and vehicles, including active vibration damping devices, shock absorbers, clutches, electrically controlled valves, and in aerospace applications. In order to increase machine efficiency and speed of movement of vehicles, newer and newer technologies are being looked for.

Currently, one of the important directions of work on improving the performance of machines and vehicle components of hydraulic subassembly is to improve the working fluid. The tests are carried out to search for new types of these liquids, such as fluids, whose properties can be altered by external influences. Therefore, works on the working fluids, whose viscosity can be varied continuously and reversibly by the electric field have a large perspective. This allows the control of devices with these liquids in a very simple way.

The main qualities of electrorheological fluids are their high yield stress and enhanced viscosity under an applied electric fields. Therefore, it is worth to study the electrorheological properties of various suspensions in order to seek out possible industrial applications wherein the suspensions of nanoparticles in the base fluid deserve particular attention.

Sheng and Wen [49] explored the interaction between nanoparticles and an electric field from the electrorheological point of view. The yield stress is one of the critical design parameters in a device containing the electrorheological liquid and has attracted substantial attention both theoretically and experimentally. Farajian et al. [50] theoretically investigated the yield stress in carbon nanotube suspensions under an electric field. On the other hand, Raykar et al. [51] reported the electrorheological properties of low-concentration Fe_2_O_3_ nanofluids prepared in ethylene glycol under the less influence of electric fields while Yin and Zhao [52] presented the recent researchers on electrorheology of various nanofiber-based suspensions, including inorganic, organic, and inorganic/organic composite nanofibers.

Viscosity of the electrorheological fluids depends primarily on the shear rate, electric field strength, and also the temperature. An important issue which could not be neglected in the course of the examination of suspension is the problem of ensuring the stability of the dispersion of the particles and their protection against agglomeration and sedimentation [53]. The long-term sedimentation causes loss of the electrorheological phenomenon despite the presence of the stimulating electric field.

Prekas et al. [54] reported the effect of temperature and surfactant concentration on the stability of electrorheology fluid prepared from zeolite particles and silicone oil.

Nanofluids may have many important applications in the industry and thus should be carefully studied, both in terms of occurrence of the electrorheological effects as well as other rheological properties. Therefore, further properties of MgAl_2_O_4_-diethylene glycol nanofluid were investigated and presented in the hereby paper.

## Methods

### Dry nanoparticles

Applied in the experiments, MgAl_2_O_4_ ceramic nanopowder was produced by Baikowski (Annecy, France, Italy). This nanopowder is commercially available as a magnesium-aluminum spinel (ID LOT: 101488). The average size of the nanoparticles which were used was 40 nm; it was measured with an X-ray diffraction technique (XRD) and scanning electron microscope (SEM).

An important application of the MgAl_2_O_4_ spinels nanopowder is its use for the preparation of the transparent ceramic [55-58]. Additional information about this process, properties of magnesium-aluminum spinel, and scanning electron microscope pictures are contained in [59].

### Sample preparation

The samples of nanofluids containing different mass concentrations of MgAl_2_O_4_ nanopowder in diethylene glycol were prepared by using a two-step method. To disperse of the MgAl_2_O_4_ nanopowder in the base fluid, the strictly defined actions were sequentially performed. The first stage was to receive the undispersed nanofluid with desired concentration of nanopowder. It was done by putting a predetermined amount of ceramic nanopowder into a glass vessel placed on an analytical balance AS 220/X (Radwag, Radom, Poland). This balance has an accuracy of measurement of 0.1 mg, and its reliability is ensured by an internal calibration. Then, using a pipette, an addition of a pure diethylene glycol (DG), manufactured by Chempur (CAS: 111-46-6, Piekary Śląskie, Poland), was used to obtain an appropriate weight of sample. In order to achieve a mechanical stirring of components, the sample was placed in a Genius 3 Vortex (IKA, Staufen, Germany) for 30 min. In view of the possibility of emergence of sedimentation of nanoparticles, the sample was inserted into an ultrasound wave bath Emmi-60HC (EMAG, Moerfelden-Walldorf, Germany) for 200 min. At this time, acting ultrasonication destroyed agglomerates of nanoparticles and prevented re-agglomeration.

A special cooling system which allowed us to maintain the temperature in the bath below 25°C was used. All nanosuspension was performed in temperature less than 25°C. More information about the ultrasound wave bath and cooling system can be found in [60]. It is worth emphasizing that other scientists also use the ultrasonication bath as a method of dispersing of nanoparticles in the base fluid [21,28,61-63]. Nanofluids prepared for measurements with this method were stable for several hours.

### Measuring system

Measurements characterizing the influence of pressure and electric field on viscosity of MgAl_2_O_4_-DG nanofluids were performed with use of a HAAKE MARS 2 rheometer (Thermo Fisher Scientific, Karlsruhe, Germany). It can be used to perform rotating or oscillating measurements. Furthermore, its modular constructions allow to adjust it for specific applications. This rheometer enables the regulation of torque from 50 nNm to 200 mNm and also the control of angular velocity from 10^−5^ to 1,500 rpm. The nozzle of the air bearing of the rheometer was connected with a compressor (FIAC Air Compressors, Bologna, Italy). Measurements were controlled using a HAAKE RheoWin Data Manager ver. 4.30.0022 (Thermo Fisher Scientific, Karlsruhe, Germany).

#### Pressure chamber

Measurements of influence of pressure on viscosity of magnesium-aluminum spinel nanopowder suspensions in diethylene glycol were performed using a HAAKE MARS 2 rheometer in conjunction with the pressure chamber D100/200 (Thermo Electron Corporation, Karlsruhe, Germany) with a PZ38 (38-mm diameter of cylinder) concentric cylindrical geometry (Thermo Electron Corporation, Karlsruhe, Germany) and a hand pump ENERPAC (ENERPAC, Menomonee Falls, WI, USA). Additionally, for controlling the temperature within the measuring system, a liquid cooling system TEF/Z48 (Thermo Electron Corporation, Karlsruhe, Germany) connected with a thermostat HAAKE Phoenix 2 (Thermo Electron Corporation, Karlsruhe, Germany) was used. Temperature of the sample was controlled with an accuracy of 0.5°C.

The experimental system consists of static and rotating parts. Construction of the cylindrical pressure chamber is divided into three main parts.

**Figure 1:**
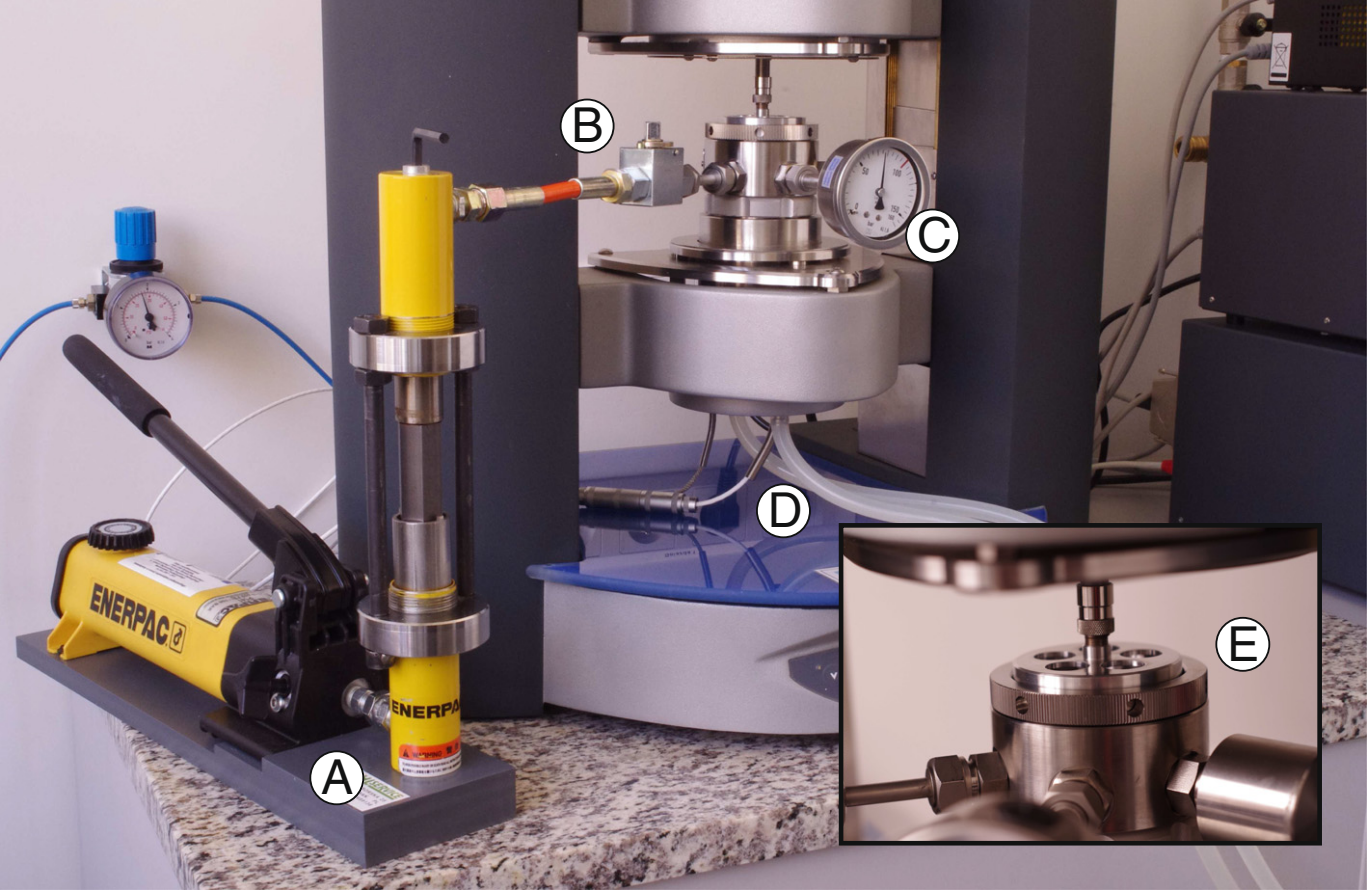
**Pressure chamber installed on HAAKE MARS 2 rheometer.(A)** hand pump ENERPAC, **(B)** valve connects the pump to the chamber, **(C)** gauge showing the current pressure in the chamber, **(D)** tubes supplying coolant thermostat, **(E)** outer magnet.

The upper part creates the outer magnet (Figure 1(E)) which is attached to the drive shaft of the measuring head of the rhometer and the upper cover which is screwed on the middle part of the pressure chamber. The central section forms the stationary measuring cell composed of the manometer (Figure 1(C)), and the rupture disk and the ball valve (Figure 1(B)) which is linked through the viton tube with a cylinder of the hand pump. The rotor and the inner magnet, which is attached to the rotor, are inserted into the interior of the measuring cell. The rotor is located between two sapphire bearings and centered by two pins. One bearing is at the bottom side of the upper lid and the second bearing is at the bottom side of the chamber. It has a much higher surface hardness and thus are resistant to the friction of the rotating rotor. The lower part of the pressure chamber includes the base with the centering pin for the rotor, and the second pin is at the top side of the rotor. Furthermore, from the bottom side of the base, a temperature sensor can be connected; it allows to control the temperature of sample during the test. The lower part is screwed on the middle part of the pressure chamber.

The magnetic coupling between the outer and inner magnet is significant. The torque acting on the rotor is transferred from the drive shaft of the measuring head of the rheometer by the magnetic coupling, causing the rotation of the rotor. The gap between the rotor and the measuring chamber has to be completely filled by the sample.

Correct calibration of the measuring system eliminates unwanted physical effects and thus allows to obtain high-quality results. Thus, the results depend only on the sample, not affected by the system, so that the viscosity of the nanofluid is measured correctly. For properly prepared measuring of geometry, a drop of oil on the sapphire bearing in the upper cover of the chamber and a few drops of the diethylene glycol on the base of the measuring cup were applied. Furthermore, the parameters of correction of friction of the rotor and the procedure of the Micro Stress Control (MSC) in the RheoWin program had to be excluded.

**Figure 2:**
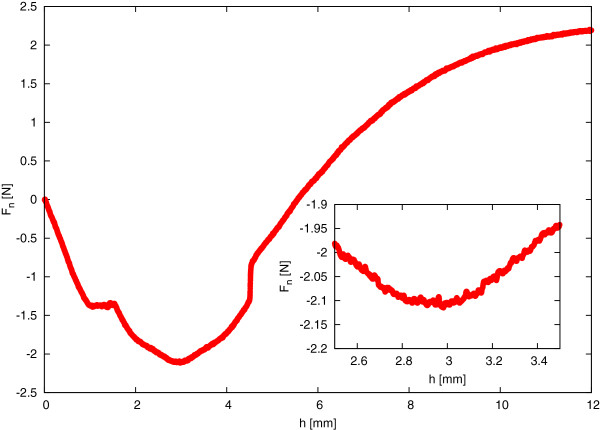
**Sample dependence of normal force (*F***_***n***_**) on the width of the measuring gap in unfilled pressure chamber.**

The first step of the correct calibration was to determine the zero point used for the rotor. The calibration of pressure chamber was performed according to strictly defined steps. Firstly, it was important to determine the optimal measuring gap, namely a distance between the outer magnet and the upper cover of the measuring cup. For the pressure chamber D100/200, the appropriate measuring gap has a value of about 3 mm. However, it was reasonable to determine the optimal gap before each series of measurements. In order to set the proper value of the gap, the dependence between a normal force acting on the rotor and a width of the measuring gap should be appointed. The value of a certain normal force thus depends on the distance among the two magnets. Figure 2 shows a sample curve which was received during the determination of the measuring gap. The rotor performed the rotation at an angular velocity of 1 rpm. The determination of the dependence of the normal force acting on the rotor as a function of the gap was performed from 0 to 12 mm, taking into account the large number of measuring points in the time of 720 s. For the values above 12 mm, the force of the magnetic field was so small that it was not enough for the transmission of the normal force acting on the rotor. The lower value of the gap means that the magnetic force was stronger.

Besides, four stages can be specified in the action of the normal force *F*_*n*_. Initially, the rotor rests on the lower sapphire bearing (the first stage). At that time, the value of normal force increases because the measuring head of the rheometer moves downwards; therefore, the magnetic coupling becomes stronger. This causes a jump of the rotor from the lower sapphire bearing to the upper sapphire bearing. At this moment, the normal force acting on the rotor decreases rapidly; it is visible on the presented curve in Figure 2. Then, the rotor is attracted by the upper bearing (the second stage); therefore, its weight is compensated by the magnetic coupling. The value of the optimal measuring gap is read when the normal force achieves its minimum value. This is both the maximum value of the magnetic force generated by the magnetic coupling. In the example presented in Figure 2, the optimal value of the gap was 2.95 mm. At this gap, the rotor levitates between the upper and lower bearing (the third stage) and the normal force has almost a constant value. Then the rotor rests on the lower sapphire bearing (the fourth stage) and the value of the normal force reaches the value of zero. Moreover, during the rotation of the rotor, the friction occurs on the sapphire bearings. The value of the friction changes depending on the normal force generated by the magnetic coupling. The lowest friction occurs when the gap is the widest (the first stage) and exactly before a jump of the rotor from the lower to the upper sapphire bearing. What is more, when the rotor levitates, the friction occurs just on the cylindrical borders of the sapphire bearings. What is interesting is that the lowest friction value is not achieved during the levitation stage, as might have been expected. This means that the friction on the cylindrical borders of the bearings has a relatively high participation in the absolute friction on the bearings.

The next step of the calibration was measuring the inertia of the rotor. It was determined for a specific measurement geometry. This function allows to specify whether there are any impurities on the surface of the rotor. In order to distinguish the statistical results, measurement was repeated five times. The final value of the inertia was calculated as an average from five measurements, and introduced to the settings of the rotor.

Subsequently, the procedure of MSC used for defining the microstrains which are generated during the operation of the rheometer was performed. The appointed value should be included for the current rotor used. The MSC values are subtracted from the results obtained during the relevant measurements.

The final step of calibration was the calculation of the friction correction parameters. For this purpose, the dependence of the friction on the sapphire bearings in the function of the rotation speed was determined. It is important to set the extent of the share rates in which the pressure chamber will be used because the same range should be applied during an appropriate measurement. Thus, it was the so-called ‘on empty’ measurement, i.e. without the sample in pressure chamber. A range of share rates from 0.01 to 1,000 s ^−1^ in time of 1,610 s was assumed. The resistances of friction depending on a rotation speed might be approximated with a mathematical equation:

(1) $$M_{e}=a\Omega^{2}+\mathrm{b\Omega}+c,$$

where *M*_*e*_ is the torque measured in empty chamber [ *μ*Nm], *Ω* is rotation speed [1/min], and *a* [ *μ*Nm/(1/*min*)^2^], *b* [ *μ*Nm/(1/*min*)], *c* [ *μ*Nm] are constant parameters of the quadratic polynomial.

**Figure 3:**
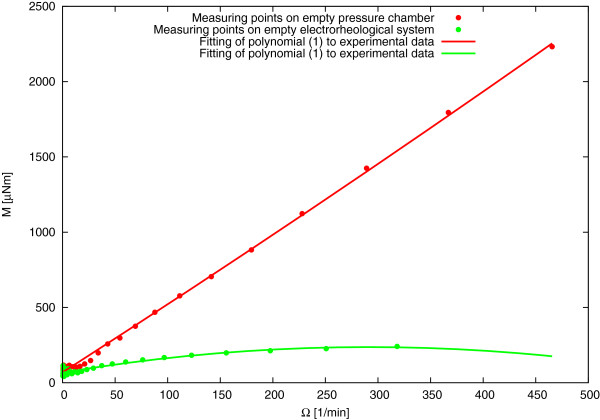
Sample on determination of friction correction parameters for pressure chamber and electrorheology system.

The parameters of the quadratic polynomial were fitted to the measurement data. Results of calculation of the friction correction parameters are presented in Figure 3. This procedure can also be used to offset the impact of the friction in bearing in electrorheological measurements so the result on the application of this procedure in electrorheology is also shown in Figure 3.

These correction parameters *a*, *b*, and *c* have to be introduced into the properties of the rotor as ‘torque correction’ in the RheoWin software. During the experiments, the torque measured by the rheometer acting on the rotor angular velocity for a given contribution is subtracted from the friction. Therefore, the measurement result recorded are calculated as

(2) $$M^{\prime }=M_{d}-\underset{M_{e}}{\underset{︸}{a\Omega^{2}+\mathrm{b\Omega}+c}},$$

where *M*_*d*_ is the actually measured torque acting on the rotor, *M* is the torque used to calculate the viscosity of the sample, taking into account the effect of friction characteristic of the measurement geometry. The described procedure can be carried out only for the rotational measurement. In the case of oscillatory measurements, it does not work; so, in using the pressure chamber or electrorheological system, it is not possible to determine the viscoelastic properties of the material.

After the calibration of the pressure chamber, its position should not be altered. The pressure chamber was filled with the hand pump. By using the automatic measuring pipette, the sample was filled with carefully into the cylinder of the hand pump. After that, the sample was pumped into the measuring chamber. These activities were repeated until the complete filling of the measuring system. The volume of the sample during the measurements was 120 cm^3^. To increase the pressure in the measuring cell, the hand pump also was used. The pressure in the experimental system was raised to the value of 7.5 MPa. Before the start of the measuring series, we checked the measuring range of PZ38 cylindrical geometry. The lower measuring range is limited to two parameters: the lowest permitted torque acting on the rotor (a) at a low shear rate is 250 *μ*Nm, measuring points collected at lower values of torque may be considered as burdened with too much uncertainty and can be rejected and (b) at high shear rates and for materials with low viscosity, the Taylor vortices can be formed, which disturbs the laminar flow in the measuring chamber. Based on theoretical considerations, Taylor [64] predicted that when the inner cylinder is rotating, there should be a certain critical frequency of rotation above which, in the flowing fluid, creates a series of regular vortices that fill the annular gap between the cylinders. Taylor not only calculated the critical frequency of the rotation, but also experimentally proved the existence of vortices. Characteristically, spiral Taylor vortices proceed the transition to turbulent motion. The axes of the vortices formed in sections of the annular gap are parallel to the primordial direction of fluid flow.

For these reasons, it is important that the shear rate range during the calibration of friction corresponded to the measuring range of the test sample with a defined viscosity. The rotation measurements under the pressure of 7.5 MPa were performed at the shear rate range from 0.01 to 1,000 s ^−1^ in the logarithmic scale. This measurements were carried out in a step procedure (50 steps) in the controlled shear rate mode (CR), wherein each data point was taken after 100 s of a constant shear rate acting on the sample in the constant temperature of 15°C±0.5°C.

The measurement of the viscosity of the MgAl_2_O_4_-DG nanofluid at a pressure of 7.5 MPa was performed at the same temperature as experiments in atmospheric pressure presented in paper [60] and the obtained results were compated.

#### Electrorheology system

**Figure 4:**
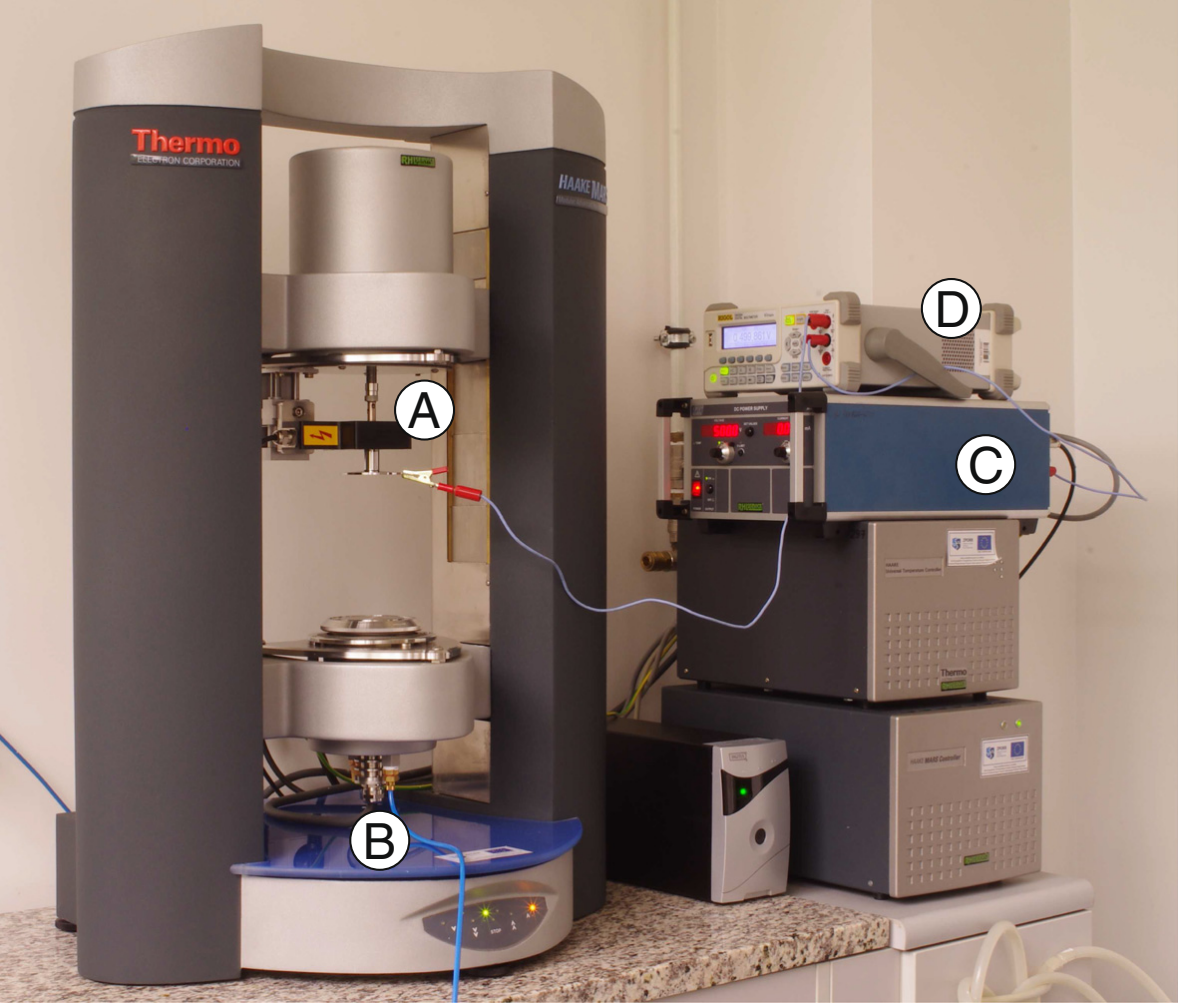
**System used to study rheological properties in electric field at position before measurement – validation of system.(A)** a transfer element connection to the rotor through a ball bearing, **(B)** compressed air supply line to the cooling system rheometer, **(C)** a voltage generator, **(D)** multimeter.

In order to perform measurements determining the influence of the electric field on the viscosity of MgAl_2_O_4_-DG nanofluids, a special electrorheology system dedicated for HAAKE MARS 2 was mounted on the rheometer. In combination with the specially adapted ER-rotors, the electrorheology system can be used for applying a high tension voltage. The abbreviation ER is derived from the name of electrorheology. Figure 4 presents the used electrorheological system before measurements.

Electrorheological measurements require the use of a special high voltage supply unit MPC 14-2000 (F.u.G. Elektronik GmbH, Rosenheim, Germany), which is shown in Figure 4(C).

The maximum allowable power in the system was 10 W at DC voltages not exceeding 2,000 V and a current of 0.01 mA (according to instruction of ThermoScientific ver. 1.0).

For the measuring head of the rheometer, an ER-adapter device for AC/DC high voltage and a high voltage plug (Thermo Fisher Scientific, Karlsruhe, Germany) were attached (Figure 4(A)). ER-adapter unit with the plug and the high-voltage supply unit (Figure 4(C)) were connected to each other *via* a high tension cable.

The measuring geometry type of PP60 (plate-plate 60-mm diameter of plate) was used. The ER-rotor was attached to the motor drive shaft of the rheometer (Figure 4(A)). The ER-rotor passes through a hole with connector in the high-voltage plug. The rotor consists of a steel and a ceramic part for isolation. An important role was played by the steel ball-bearing, used to transition the high voltage onto a rotating steel shaft of the rotor, which was insulated from the rest of the system by the mentioned ceramic.

The voltage was transmitted thanks to the two contacts situated in a hole of the high-voltage plug. These contacts were in touch with the steel bearing of the rotor. Therefore, the rotational movement of the ER-rotor was related with the occurrence of a certain friction, which must be taken into account and corrected, so the measured values of viscosity are affected by the lowest error.

Additionally, the rheometer and the high-voltage supply unit were connected to each other *via* a grounding cable, which is designed to protect microelectronics of the rheometer against damage. Moreover, for the rheometer, it was connected to an air hose (Figure 4(B)), which supplied air with compressor situated in the laboratory.

The physical processes taking place in the assembled system can lead to the formation of electrorheological effects, which is an equivalent to the formation of agglomerates in the test sample (possibly under the influence of the Coulomb force) and then the formation of chains of agglomerates.

**Figure 5:**
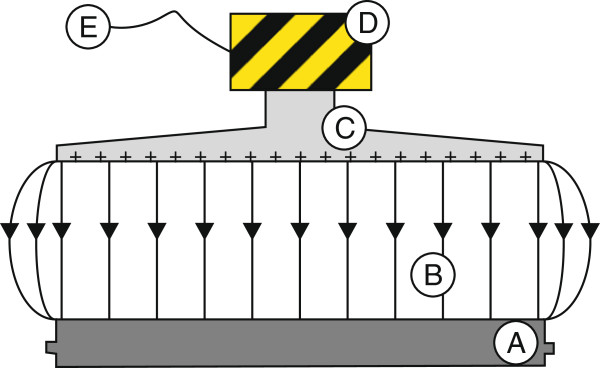
**Diagram of electric field in mounted electrorheological system.(A)** Stable lower plate, **(B)** field lines, **(C)** ER-rotor, **(D)** ER-adapter, **(E)** positive electrode connected to a high-voltage power supply.

**Figure 6:**
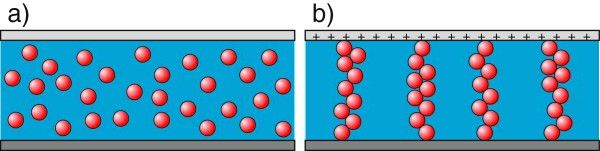
**Position of particles in diphase electrorheological fluid.(a)** In the absence of an electric field; **(b)** in the presence of an electric field.

The positive electrode (Figure 5(E)), connecting the power supply unit with the high-voltage plug (Figure 5(D)), creates an electric field on the rotor (Figure 5(C)). Due to the low width of the gap and a relatively large area of measuring plate, it can be assumed that the force lines of electric field are perpendicular to the measurement plates (Figure 5(B)). The positive electrode ‘receives’ free electrons from the sample (Figure 5(A)), leaving an electron hole in their place. If the remaining particles are charged, action of the Coulomb force causes them to start to move in the direction of the electrode. In this way, in the structure of the test sample, the chains of agglomerates may be formed (Figure 6).

The same as in the case of pressure measurements before each test of the sample, the calibration of the entire system was performed. Firstly, the zero point for used ER-rotor was determined. During this procedure, the rotor was in contact with the bottom measuring plate. This operation was performed in order to obtain the repeatable gap. For the ER-rotor, the width of the gap was not determined, it was constant and equal to 1 mm.

Subsequently, the inertia was measured using the automatic function ‘Device Manager’, in the same way as that used for the pressure measurements described above. Wherein, the ball-bearing was not in contact with the hole of the insulted high-voltage plug. Thereby, the additional friction has not occurred. This was important because in this case, only the parameters of the ER-rotor is considerable. Then, the procedure of MSC, namely a reduction of microstrains generated in the engine of the rheometer at a torque value 50 nNm was performed, also in the same manner as that used for pressure measurements. This procedure was performed in the same way as inertia thus without contact between the bearing and the high-voltage plug.

At the end of calibration of the electrorheology system, the friction correction was carried out. The whole procedure was the same as in the case of pressure measurements (described in ‘Pressure chamber’), although friction was derived from various elements of used geometry (friction of the sapphire bearing within the pressure chamber and friction of the ball bearing in electrorheology).

In addition, before the start of the measuring series, the measuring range of ER-geometry was checked. However, it is not suggested in the specification of the ER-rotor PP60, whereby it is recommended rejection of values less than 50 *μ*Nm. The measurement data, for which the moment of the force was less than 100 *μ*Nm, were rejected.

The original electrorheological system designed for HAAKE MARS 2 is not equipped with any diagnostic tool allowing to determine whether the system is working properly. It is not possible to check whether the sample is actually located in an electric field or not. Furthermore, before each series of measurements, the multimeter Rigol DM 3064 (Rigol, Beijing, China) was pinned to the rotor (Figure 4(D)). In this way, it was checked whether the voltage is correctly supplied to the rotor, and we are sure that each sample was measured in an electric field.

After completing all the calibration steps and finding that they were all carried out properly, the measurement of the earlier prepared sample was started. The sample of nanofluid with an automatic pipette on the lower measurement plate was applied, volume of the sample was 2.7 cm^3^. On the power supply unit, the desirable voltage was set, and then it was turned, thereby the voltage to the rotor was brought.

The first measurement was performed in the absence of voltage. Afterward, the sample has been tested for the following values of voltage: 500, 1,000, 1,500, and 2,000 V.

The measurements of dynamic viscosity curves were performed in a step procedure in the CR mode at the shear range from 1 to 1,000 s ^−1^ in the logarithmic scale. Each of the 30 steps took 100 s, wherein the value of the shear rate acting on the sample at that time was constant. The measurement points were collected on the basis of the results obtained in the last 3 s of a single step. In the course of measurements, it was not possible to maintain a constant temperature because an imposed mode of operation of the assembled system has made it impossible.

A thixotrophic behavior was observed upon measurement in CR mode performed in three steps. First sample was measured with increase of shear rate from 1 to 1,000 s ^−1^ in a time of 600 s. The second step was shearing the sample with a constant shear rate of 1,000 s ^−1^ used for 600 s. The third stage of experiment was the measurement with shear rate decreasing from 1,000 to 1 s ^−1^ in 600 s.

In view of the fact that the measuring geometry was an air-cooled system, it was not possible to achieve a constant temperature during the measurements. The system was purged with air at room temperature, and the lab has efficient air conditioning system. Nevertheless, temperature spread reached 1.5°C.

## Results and discussion

### Pressure measurement

**Figure 7:**
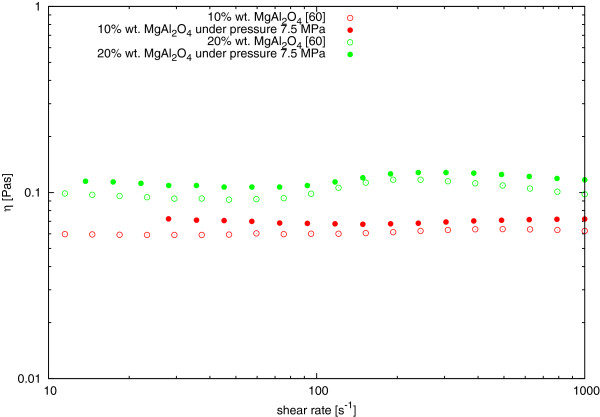
**Comparison of dynamic viscosity of MgAl**_**2**_**O**_**4**_**-DG nanofluids in normal conditions [**[60]**] and under a pressure of 7.5 MPa.**

A study to determine the dynamic viscosity curve of MgAl_2_O_4_-DG nanofluid under anisotropic high pressure was conducted. The experiment was performed on two samples of different mass concentrations of nanoparticles in nanofluid, namely 10 and 20 wt.%. The results of the measurements are presented in Figure 7, where there is a comparison to the results obtained during the experiment in the pressure chamber and under normal conditions, described in [60], full circles present viscosity values with a pressure of 7.5 MPa, while empty circles present those in normal conditions.

The increase in viscosity of the material subjected to anisotropic pressure of 7.5 MPa was in the range from 10.04% to 22.04% for the 10% mass concentration of the nanoparticles in suspension. The suspension of 20 wt.% concentration of nanoparticles increase in dynamic viscosity from 6.19% to 19.54% in the tested range of shear rates. The test results clearly show that pressure affects on the dynamic viscosity of examined nanofluids, causes it to rise, but does not change the nature of the viscosity curve. The effect of maximum of viscosity curve for some shear rate could be seen and described in [60]. This demonstrates that this effect does not depend on the measurement method, or the nature of the measuring geometry used.

### Electrorheology

**Figure 8:**
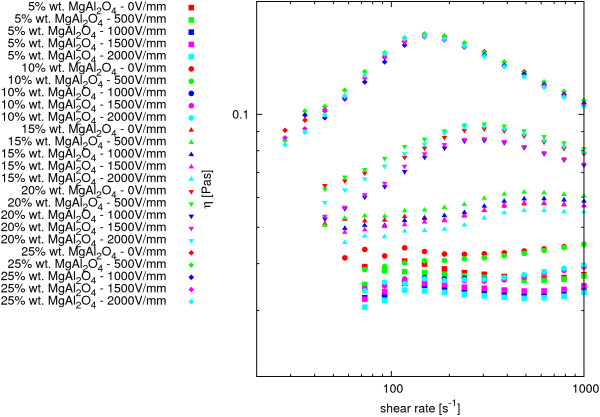
**Comparison of dynamic viscosity of MgAl**_**2**_**O**_**4**_**-DG nanofluids at various intensities of electric field in temperature*(22.5±1.5)°*C.** Different types of points correspond to different mass concentrations of nanoparticles in nanofluid; colors indicate different intensities of electric field.

A study on the impact of the applied electric field on the dynamic viscosity of MgAl_2_O_4_-DG nanofluids was performed. Experiments were conducted in the electric field intensity from 0 to 2,000 V/mm using the same measurement process used to study the material viscosity curves under normal conditions presented in [60]. The experimental results are summarized in Figure 8; various colors indicate the results for each value of the electric field, and the different types of points correspond to different mass concentrations of nanoparticles in nanosuspension.

Reasons for differences between the results of measurements of dynamic viscosity of nanofluids in the same mass concentration of nanoparticles at various values of the electric field should be sought in imperfection of measurement system, in which it is impossible to make measurements at constant temperature. As previously described, an air-cooled system can work only in room temperature; a cooling system is effective at temperatures higher than 40°C. In the Laboratory of Biophysics at Rzeszów University of Technology, measurements were conducted in an operational air conditioning system, but in spite of this, there is a fluctuation in air temperature. The measurement data were collected in temperatures ranging from 21°C to 24°C. Based on this information, it can be assumed that the electric field does not affect the dynamic viscosity of the test material in the test range of electric field.

**Figure 9:**
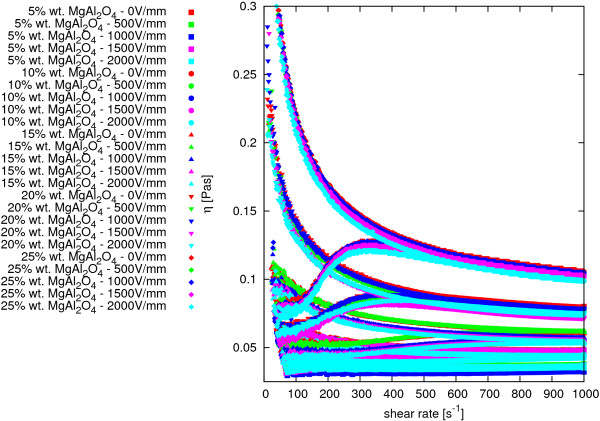
**Comparison of thixotropic properties of MgAl**_**2**_**O**_**4**_**-DG nanofluids at various intensities of electric field in temperature*(22.5±1.5)°*C.** Different types of points correspond to different mass concentrations of nanoparticles in nanofluid; colors indicate different intensities of electric field.

Other tests were conducted on MgAl_**2**_**O**_**4**_**-DG nanofluid to study the effect of the electric field on a substance’s thixotropic behavior. The method of measurement was analogous to the measuring of thixotropy under normal conditions presented in [**[60]. The results of these measurements are summarized in Figure 9; various colors indicate the results for each value of the electric field, and the different types of points correspond to different mass concentrations of nanoparticles in nanosuspension.

Presented data show that an applied electric field does not affect the thixotropic behavior of the tested materials; any differences are due to the lack of capacity to perform measurements at a constant temperature.

MgAl_**2**_**O**_**4**_, in the macroscopic scale, is a material used for the production of transparent ceramics, which can be used as an insulator. It was to be expected that nanoparticles of this material are non-polar and the effects of electrorheological properties may not be noticeable. However, due to the fact that repeatedly observed change in physical properties of materials at the nanoscale, the material should be examined for such behavior.

## Conclusions

The paper presents new experimental data on rheology of MgAl_**2**_**O**_**4**_-DG nanofluids.

Samples were measured under the anisotropic pressure of 7.5 MPa to determine viscosity curves in these conditions. It showed an increase in dynamic viscosity compared to the results obtained at atmospheric pressure, which did not show a change in the nature of the viscosity curve.

A study has also been conducted on viscosity curves and thixotropic properties for different mass concentrations of nanoparticles in nanofluid, depending on the intensity of the applied electric field. There was no influence of the electric field on dynamic viscosity and thixotropic properties of the tested materials.

The paper demonstrates that the electric field has no effect on the rheological properties of the MgAl_**2**_**O**_**4**_-DG nanofluids. This is a very valuable information for potential industrial applications because it shows that one can use these nanofluids in the presence of an electric field without worrying about changing the viscosity of the material in these conditions.

Despite the use in the studies of three different types of measuring geometries (a) coaxial cylinders in pressure chamber, (b) plate-plate geometry in electrorheological study, and (c) double cone geometry in experiments under normal conditions [60], the character of dynamic viscosity curve for the tested material remains unchanged. On the viscosity curves, there can still be observed areas in which the viscosity decreases, increases, and decreases again. Thus, it was demonstrated that, beyond any doubt, this behavior does not depend on the type of measurement geometry used.

## Competing interests

The authors declare that they have no competing interests.

## Authors’ contributions

Gż planned the measurements, performed the samples, conducted the study, has made the processing and analysis of data, took an active part in the discussion of the results and preparation of the manuscript, and coordinated the research. JG performed the samples, conducted the study, and took an active part in the discussion of the results and preparation of the manuscript. AW has prepared materials for research and took an active part in discussions of the results and preparation of the manuscript. MC took an active part in discussions of the results. All authors read and approved the final manuscript.

## References

[B1] Eijkel JCT van den Berg A **Nanofluidics: what is it and what can we expect from it?** Microfluidics Nanofluidics 2005 9 3 249 267 10.1007/s10404-004-0012-9

[B2] Taylor R Coulombe S Otanicar T Phelan P Gunawan A Lv W Rosengarten G Prasher R Tyagi H **Small particles, big impacts: a review of the diverse applications of nanofluids** J Appl Phys 2013 9 1 011301 10.1063/1.4754271

[B3] Nie S Xing Y Kim GJ Simons JW **Nanotechnology applications in cancer** Annu Rev Biomed Eng 2007 9 1 257 288 10.1146/annurev.bioeng.9.060906.152025 17439359

[B4] Thomas S Balakrishna Panicker Sobhan C **A review of experimental investigations on thermal phenomena in nanofluids** Nanoscale Res Lett 2011 9 1 377 10.1186/1556-276X-6-377 21711918 PMC3211470

[B5] Yu W Xie H Li Y Chen L **Experimental investigation on thermal conductivity and viscosity of aluminum nitride nanofluid** Particuology 2011 9 2 187 191 10.1016/j.partic.2010.05.014

[B6] Pastoriza-Gallego MJ Lugo L Legido JL Piñeiro MM **Enhancement of thermal conductivity and volumetric behavior of Fe**_ ** *x* ** _**O**_ ** *y* ** _** nanofluids** J Appl Phys 2011 9 1 014309 10.1063/1.3603012

[B7] Pastoriza-Gallego M Lugo L Legido J Piñeiro M **Thermal conductivity and viscosity measurements of ethylene glycol-based Al**_ **2** _**O**_ **3** _** nanofluids** Nanoscale Res Lett 2011 9 1 221 10.1186/1556-276X-6-221 21711737 PMC3211279

[B8] Martin-Gallego M Verdejo R Khayet M Ortiz de Zarate JM Essalhi M Lopez-Manchado MA **Thermal conductivity of carbon nanotubes and graphene in epoxy nanofluids and nanocomposites** Nanoscale Res Lett 2011 9 1 610 10.1186/1556-276X-6-610 22133094 PMC3285700

[B9] Baby TT Ramaprabhu S **Experimental investigation of the thermal transport properties of a carbon nanohybrid dispersed nanofluid** Nanoscale 2011 9 2208 2214 10.1039/c0nr01024c 21455535

[B10] Kleinstreuer C Feng Y **Experimental and theoretical studies of nanofluid thermal conductivity enhancement: a review** Nanoscale Res Lett 2011 9 1 229 10.1186/1556-276X-6-229 21711739 PMC3211287

[B11] Sergis A Hardalupas Y **Anomalous heat transfer modes of nanofluids: a review based on statistical analysis** Nanoscale Res Lett 2011 9 1 391 10.1186/1556-276X-6-391 21711932 PMC3211485

[B12] Mallick SS Mishra A Kundan L **An investigation into modelling thermal conductivity for alumina-water nanofluids** Powder Technol 2013 9 234 244

[B13] Hirota K Sugimoto M Kato M Tsukagoshi K Tanigawa T Sugimoto H **Preparation of zinc oxide ceramics with a sustainable antibacterial activity under dark conditions** Ceramics Int 2010 9 2 497 506 10.1016/j.ceramint.2009.09.026

[B14] Zhang L Jiang Y Ding Y Povey M York D **Investigation into the antibacterial behaviour of suspensions of ZnO nanoparticles (ZnO nanofluids)** J Nanoparticle Res 2007 9 3 479 489 10.1007/s11051-006-9150-1

[B15] Timofeeva EV Routbort JL Singh D **Particle shape effects on thermophysical properties of alumina nanofluids** J Appl Phys 2009 9 1 014304 10.1063/1.3155999

[B16] Warrier P Teja A **Effect of particle size on the thermal conductivity of nanofluids containing metallic nanoparticles** Nanoscale Res Lett 2011 9 1 247 10.1186/1556-276X-6-247 21711761 PMC3211308

[B17] Feng Y Yu B Xu P Zou M **The effective thermal conductivity of nanofluids based on the nanolayer and the aggregation of nanoparticles** J Phys D: Appl Phys 2007 9 10 3164 10.1088/0022-3727/40/10/020

[B18] Pastoriza-Gallego MJ Casanova C Legido JL Piñeiro MM **CuO in water nanofluid: influence of particle size and polydispersity on volumetric behaviour and viscosity** Fluid Phase Equilibria 2011 9 1-2 188 196 10.1016/j.fluid.2010.10.015

[B19] Heine DR Petersen MK Grest GS **Effect of particle shape and charge on bulk rheology of nanoparticle suspensions** J Chem Phys 2010 9 18 184509 10.1063/1.3419071

[B20] Einstein A **Eine neue bestimmung der molekul-dimension (a new determination of the molecular dimensions)** Annalen der Physik 1906 9 2 289 306

[B21] Li Y Zhou J Tung S Schneider E Xi S **A review on development of nanofluid preparation and characterization** Powder Technol 2009 9 2 89 101 10.1016/j.powtec.2009.07.025

[B22] Chen H Ding Y Tan C **Rheological behaviour of nanofluids** New J Phys 2007 9 10 367 10.1088/1367-2630/9/10/367

[B23] Mackay ME Dao TT Tuteja A Ho DL Van Horn B Kim H-C Hawker CJ **Nanoscale effects leading to non-Einstein-like decrease in viscosity** Nat Mater 2003 9 11 762 766 10.1038/nmat999 14566332

[B24] Zubarev ER **Nanoparticle synthesis any way you want it** Nat Nanotechnol 2013 9 396 397 10.1038/nnano.2013.109 23728073

[B25] Chang M-H Liu H-S Tai CY **Preparation of copper oxide nanoparticles and its application in nanofluid** Powder Technol 2011 9 1-3 378 386 10.1016/j.powtec.2010.11.022

[B26] Yu W Xie H **A review on nanofluids: preparation, stability mechanisms, and applications** J Nanomaterials 2012 9 435873

[B27] Fedele L Colla L Bobbo S Barison S Agresti F **Experimental stability analysis of different water-based nanofluids** Nanoscale Res Lett 2011 9 1 300 10.1186/1556-276X-6-300 21711817 PMC3211367

[B28] Chung SJ Leonard JP Nettleship I Lee JK Soong Y Martello DV Chyu MK **Characterization of ZnO nanoparticle suspension in water: effectiveness of ultrasonic dispersion** Powder Technol 2009 9 1 2 75 80

[B29] Chen H Ding Y Lapkin A Fan X **Rheological behaviour of ethylene glycol-titanate nanotube nanofluids** J Nanoparticle Res 2009 9 1513 1520 10.1007/s11051-009-9599-9

[B30] Tamjid E Guenther BH **Rheology and colloidal structure of silver nanoparticles dispersed in diethylene glycol** Powder Technol 2010 9 1-2 49 53 10.1016/j.powtec.2009.08.022

[B31] żyła G Witek A Cholewa M **Viscosity of diethylene glycol-based Y**_ **2** _**O**_ **3** _** nanofluids** J Exp Nanosci (IN PRESS) 2013 DOI: 10.1080/17458080.2013.841999, http://dx.doi.org/10.1080/17458080.2013.841999 10.1186/1556-276X-9-170 PMC3986465 24712490

[B32] Hu P Shan W-L Yu F Chen Z-S **Thermal conductivity of AlN - ethanol nanofluids** Int J Thermophys 2008 9 6 1968 1973 10.1007/s10765-008-0529-3

[B33] żyła G Cholewa M Witek A Plog JP Lehmann V Oerther T Dieter G **Viscosity of suspensions of yttrium oxide (Y**_ **2** _**O**_ **3** _**) nanopowder in ethyl alcohol** J Nanosci Nanotechnol 2012 9 12 8920 8928 10.1166/jnn.2012.6716 23447939

[B34] Bridgman PW **The viscosity of liquids under pressure** Proc Nat Acad Sci 1925 9 10 603 606 10.1073/pnas.11.10.603 16587047 PMC1086169

[B35] Barnes HA 2000 Wales: The University of Wales Institute of Non-Newtonian Fluid Mechanics

[B36] Schmelzer JWP Zanotto ED Fokin VM **Pressure dependence of viscosity** J Chem Phys 2005 9 7 074511 10.1063/1.1851510 15743258

[B37] Wonham J **Effect of pressure on the viscosity of water** Nature 1967 9 5105 1053 1054 10.1038/2151053a0

[B38] Bett KE Cappi JB **Effect of pressure on the viscosity of water** Nature 1965 9 4997 620 621 10.1038/207620a0

[B39] Horne RA Johnson DS **The viscosity of water under pressure** J Phys Chem 1966 9 7 2182 2190 10.1021/j100879a018

[B40] Stanley EM Batten RC **Viscosity of water at high pressures and moderate temperatures** J Phys Chem 1969 9 5 1187 1191 10.1021/j100725a002

[B41] Först P Werner F Delgado A **The viscosity of water at high pressures - especially at subzero degrees centigrade** Rheologica Acta 2000 9 6 566 573 10.1007/s003970000114

[B42] Grimes CE Kestin J Khalifa HE **Viscosity of aqueous potassium chloride solutions in the temperature range 25-150.degree.C and the pressure range 0-30 MPa** J Chem Eng Data 1979 9 2 121 126 10.1021/je60081a007

[B43] Oliveira CMBP Wakeham WA **The viscosity of five liquid hydrocarbons at pressures up to 250 MPa** Int J Thermophys 1992 9 5 773 790 10.1007/BF00503906

[B44] Pastoriza-Gallego MJ Casanova C Paramo R Barbes B Legido JL Pineiro MM **A study on stability and thermophysical properties (density and viscosity) of Al**_ **2** _**O**_ **3** _** in water nanofluid** J Appl Phys 2009 9 6 064301 0643018 10.1063/1.3187732

[B45] Cabaleiro D Pastoriza-Gallego MJ Gracia-Fernández C Pineiro MM Lugo L **Rheological and volumetric properties of TiO**_ **2** _**-ethylene glycol nanofluids** Nanoscale Res Lett 2013 9 1 1 13 10.1186/1556-276X-8-1 23763850 PMC3771411

[B46] Winslow WM **Induced fibration of suspensions** J Appl Phys 1949 9 12 1137 1140 10.1063/1.1698285

[B47] Parthasarathy M Klingenberg DJ **Electrorheology: mechanisms and models** Mater Sci Eng R: Rep 1996 9 2 57 103 10.1016/0927-796X(96)00191-X

[B48] Hao T **Electrorheological suspensions** Adv Colloid Interface Sci 2002 9 1-3 1 35 10.1016/S0001-8686(01)00045-8 12027018

[B49] Sheng P Wen W **Electrorheology: statics and dynamics** Solid State Commun 2010 9 1023 1039 10.1016/j.ssc.2010.01.020

[B50] Farajian AA Pupysheva OV Schmidt HK Yakobson BI **Polarization, energetics, and electrorheology in carbon nanotube suspensions under an applied electric field: an exact numerical approach** Phys Rev B 2008 9 7 205432

[B51] Raykar VS Sahoo SK Singh AK **Giant electrorheological effect in Fe**_ **2** _**O**_ **3** _** nanofluids under low dc electric fields** J Appl Phys 2010 9 3 034306 0343065 10.1063/1.3462445

[B52] Yin J Zhao X **Electrorheology of nanofiber suspensions** Nanoscale Res Lett 2011 9 1 1 17 10.1186/1556-276X-6-256 PMC3211318 21711790

[B53] Witharana S Palabiyik I Musina Z Ding Y **Stability of glycol nanofluids - the theory and experiment** Powder Technol 2013 9 72 77

[B54] Prekas K Shah T Soin N Rangoussi M Vassiliadis S Siores E **Sedimentation behaviour in electrorheological fluids based on suspensions of zeolite particles in silicone oil** J Colloid Interface Sci 2013 9 58 64 23623409 10.1016/j.jcis.2013.03.040

[B55] Tsukuma K **Transparent MgAl**_ **2** _**O**_ **4** _** spinel ceramics produced by hip post-sintering** Nippon Seramikkusu Kyokai gakujutsu ronbunshi (J Ceramic Soc Jpn) 2006 9 1334 802 806 10.2109/jcersj.114.802

[B56] Wang SF Zhang J Luo DW Gu F Tang DY Dong ZL Tan GEB Que WX Zhang TS Li S Kong LB **Transparent ceramics: processing, materials and applications** Prog Solid State Chem 2013 9 1-2 20 54 10.1016/j.progsolidstchem.2012.12.002

[B57] Li J-G Ikegami T Lee J-H Mori T **Fabrication of translucent magnesium aluminum spinel ceramics** J Am Ceramic Soc 2000 9 11 2866 2868

[B58] Zhang J Lu T Chang X Wei N Xu W **Related mechanism of transparency in MgAl**_ **2** _**O**_ **4** _** nano-ceramics prepared by sintering under high pressure and low temperature** J PhysD: Appl Phys 2009 9 5 052002 10.1088/0022-3727/42/5/052002

[B59] żyła G Cholewa M Witek A **Dependence of viscosity of suspensions of ceramic nanopowders in ethyl alcohol on concentration and temperature** Nanoscale Res Lett 2012 9 1 412 10.1186/1556-276X-7-412 22824064 PMC3502473

[B60] żyła G Cholewa M Witek A **Rheological properties of diethylene glycol-based MgAl**_ **2** _**O**_ **4** _** nanofluids** RSC Adv 2013 9 18 6429 6434 10.1039/c3ra40187a

[B61] Hwang Y Lee J-K Lee J-K Jeong Y-M Cheong S-i Ahn Y-C Kim SH **Production and dispersion stability of nanoparticles in nanofluids** Powder Technol 2008 9 2 145 153 10.1016/j.powtec.2007.11.020

[B62] Duan F Wong T Crivoi A **Dynamic viscosity measurement in non-Newtonian graphite nanofluids** Nanoscale Res Lett 2012 9 1 360 10.1186/1556-276X-7-360 22747975 PMC3464759

[B63] Pastoriza-Gallego MJ Lugo L Cabaleiro D Legido JL Pineiro MM **Thermophysical profile of ethylene glycol-based ZnO nanofluids** J Chem Thermodynamics (IN PRESS) 2013-101016201307002. http://dx.doi.org/10.1016/j.jct.2013.07.002

[B64] Taylor GI **Stability of a viscous liquid contained between two rotating cylinders** Philos Trans R Soc Lond A, Containing Papers Math Phys Character 1923 9 605-615 289 343

